# Cinnamic Acid Derivatives as Potential Multifunctional Agents in Cosmetic Formulations Used for Supporting the Treatment of Selected Dermatoses

**DOI:** 10.3390/molecules29235806

**Published:** 2024-12-09

**Authors:** Małgorzata Kabat, Justyna Popiół, Agnieszka Gunia-Krzyżak

**Affiliations:** 1 Department of Bioorganic Chemistry, Chair of Organic Chemistry, Faculty of Pharmacy, Jagiellonian University Medical College, 30-688 Krakow, Poland; malgorzata.zofia.kabat@doctoral.uj.edu.pl; 2 Department of Pharmaceutical Biochemistry, Faculty of Pharmacy, Jagiellonian University Medical College, 30-688 Krakow, Poland; justyna.popiol@uj.edu.pl

**Keywords:** cinnamic acid, cinnamic acid derivatives, cosmetics, antioxidant, anti-inflammatory, hyperpigmentation, dermatoses

## Abstract

Cinnamic acid and its natural derivatives were primarily used in cosmetics as fragrance materials as well as skin and hair conditioners. Nowadays, not only natural but also synthetic cinnamic acid derivatives are used as active ingredients of cosmetic formulations. They still serve as fragrance ingredients but also as active ingredients supporting the treatment of selected dermatoses such as acne vulgaris, atopic dermatitis, and hyperpigmentation. They are also commonly used in anti-aging cosmetic formulations. On the other hand, several cinnamic acid derivatives used as fragrances in cosmetic products are classified as potential allergens which can cause contact dermatitis. The main mechanisms of action proved for various cinnamic acid derivatives include antioxidant, antimicrobial, anti-inflammatory, and antimelanogenic properties. Most commonly used cinnamic acid derivatives in cosmetics products are hydroxy acids such as ferulic acid, caffeic acid, *p*-coumaric acid, and sinapic acid. Chemical synthesis led to several modified acids, esters, and amides, which also showed the potential to be used in cosmetic formulations.

## 1. Introduction

Skin disorders such as acne vulgaris, atopic dermatitis, post-inflammatory hyperpigmentation, and contact dermatitis belong to the most prevalent dermatoses, significantly affecting patients’ quality of life [1,2]. Although these conditions are characterised by diverse aetiologies and mechanisms, they share a common underlying factors: an association with oxidative stress, colonisation by microorganisms, and inflammatory processes, which play pivotal roles in their pathogenesis [3].

Acne vulgaris is a dermatological condition of the pilosebaceous unit. This condition arises from a combination of factors, including excessive sebum production, abnormal keratinisation, bacterial colonisation, and inflammation. A key player in its pathogenesis is the Gram-positive anaerobic bacterium *Cutibacterium acnes*, which predominantly colonises the pilosebaceous units of human skin [4]. The immune response to *C. acnes* is triggered by fatty acids and chemotactic factors produced by the bacteria, which activate Toll-like receptor 2 (TLR2) on keratinocytes and immune cells. This activation initiates inflammatory cascades, leading to the production of pro-inflammatory cytokines [5]. Additionally, oxidative stress plays a pivotal role in acne pathogenesis. Increased levels of reactive oxygen species (ROS) promote squalene peroxidation, which exacerbates inflammation and damages skin cells. Another contributor to the inflammatory environment in acne is the enzymatic activity of 5-lipoxygenase (5-LOX) [6,7].

Atopic dermatitis (AD) is a chronic and recurrent skin condition characterised by intense itching, dryness, and recurrent skin changes. The pathogenesis of AD involves a complex interplay between immune dysregulation and skin barrier dysfunction. A key factor in skin barrier dysfunction is the mutation in the gene encoding filaggrin (FLG), a structural protein critical for maintaining the skin’s integrity and hydration. Mutations in FLG impair barrier function, leading to increased trans-epidermal water loss (TEWL) and increased sensitivity to allergens and irritants. Excessive colonisation by *Staphylococcus aureus* further exacerbates inflammation through the production of superantigens, which stimulate the release of pro-inflammatory cytokines and impair skin homeostasis [8]. Recent studies have highlighted the importance of the skin microbiota in regulating the immune response and maintaining skin barrier integrity, suggesting a dynamic relationship between microbial communities and the progression of atopic dermatitis [9].

Contact dermatitis, characterised by redness, itching, and swelling, develops after the skin is exposed to an irritant or allergen. In irritant contact dermatitis, the skin is directly damaged by a substance, leading to the release of pro-inflammatory cytokines. This form of dermatitis is typically immediate and non-immunologic in nature, as it arises from direct chemical or physical damage to the skin barrier [10]. In contrast, allergic contact dermatitis involves an immune-mediated delayed hypersensitivity reaction. Upon exposure to an allergen, the immune system activates T-cells, which recognise the allergen as a threat. This triggers the release of cytokines that amplify inflammation, causing symptoms at the site of contact [11].

Post-inflammatory hyperpigmentation (PIH) is a common consequence of inflammatory conditions such as acne vulgaris, atopic dermatitis, or contact dermatitis. PIH is linked to the process of melanogenesis, which involves the synthesis and deposition of melanin pigment in keratinocytes by melanocytes located in the basal layer of the epidermis. Melanin production is a complex process regulated by several enzymes, including tyrosinase and tyrosinase-related proteins 1 and 2, which play critical roles in catalysing the chemical reactions required for melanin synthesis [12,13].

**Figure 1:**
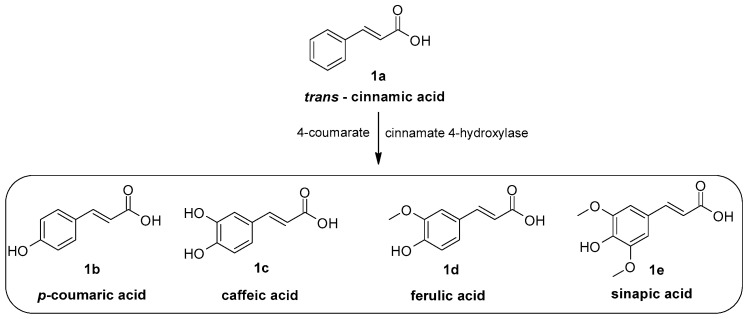
Hydroxycinnamic acids—a simplified diagram of the shikimic acid pathway (prepared according to [26]).

**Figure 3:**
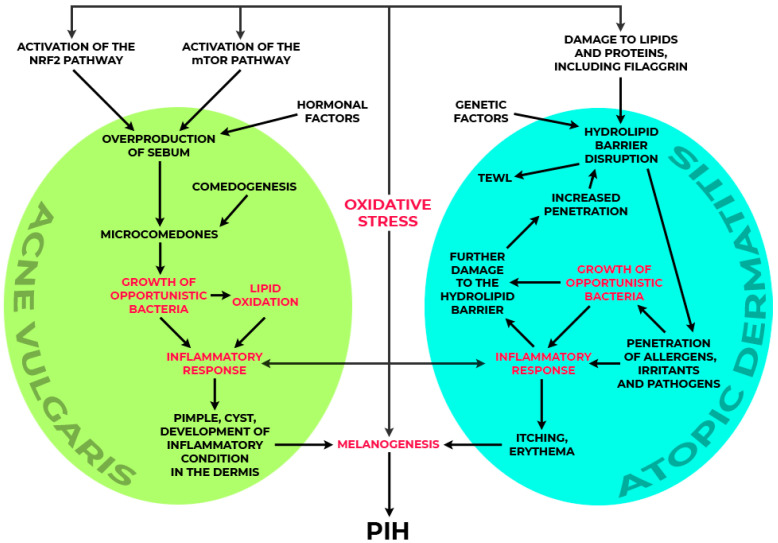
Potential therapeutic targets of cinnamic acid derivatives in acne vulgaris and atopic dermatitis (prepared according to [31,85,86,87]). The molecular and biological targets highlighted in red (lipid oxidation, microbial growth, inflammatory response, and melanogenesis) illustrate the critical points at which cinnamic acid derivatives may exert therapeutic effects in acne vulgaris, atopic dermatitis, and post-inflammatory hyperpigmentation (PIH). By modulating oxidative stress, inflammation, and microbial growth, cinnamic acid derivatives offer a comprehensive action in supporting the treatment and alleviating the symptoms of these dermatological conditions.

Cinnamic acid and its derivatives occur widely in plants, where they are synthesised in the shikimic acid pathway (Figure 1). The precursors of this pathway are tyrosine and phenylalanine; cinnamic acid is a key intermediate of this pathway and a substrate for the synthesis of flavonoids, lignins, tannins, stilbenes, anthocyanins, and coumarins, which play roles in the growth, reproduction, and protection of plants from pathogens [14,15]. Cinnamic acid (INCI: Cinnamic acid, Figure 1, **1a**), is a monocarboxylic acid that consists of acrylic acid with a phenyl substituent in the 3-position. It gets its name from the spice cinnamon (bark of *Cinnamomum zeilanicum*). It may form esters with acids containing a hydroxyl group, lipids, or sugars and amides with aromatic and aliphatic amines [16]. Cinnamic acid naturally occurs in either the *cis* or *trans* form, although the *trans* form is predominant due to its greater stability compared to the *cis* form [17]. Apart from cinnamic acid, an important group of its natural derivatives already used in cosmetics constitutes its hydroxyl derivatives such as 4-hydroxycinnamic acid (*p*-coumaric acid, INCI: Hydroxycinnmiac acid, Figure 1, **1b**), 3,4-dihydroxycinnamic acid (INCI: Caffeic acid, Figure 1, **1c**), 4-hydroxy-3-methoxycinnamic acid (INCI: Ferulic acid, Figure 1, **1d**), and 3,5-dimethoxy-4-hydroxycinnamic acid (INCI: Sinapic acid, Figure 1, **1e**). *p*-coumaric acid is commonly found in plants, including ginseng [18] and *Camellia* seed oils [19]. Ferulic acid occurs in a significant amount in wheat bran, tomatoes, and cucurbit [20], as well as several herbs spices like thyme, sage, marjoram, and rosemary [21]. Sinapic acid is a compound commonly found in plants such as rye, mustard, and berries. It is also a component of propolis [22]. Caffeic acid is one of the most common phenolic acids found in plants and mushrooms [23]. One of the best sources of caffeic acid, besides coffee beans, is rosemary (*Rosmarinus officinalis*). Caffeic acid is present in the seeds of plants belonging to the *Camellia* genus [19] as well as in thyme [24] and mint [25].

Cinnamic acid and its derivatives obtained from natural sources were primarily used as fragrance materials as well as skin and hair conditioners. Hydroxycinnamic acids and their derivatives exhibit a range of beneficial cosmetic and dermatological properties, making them valuable in cosmetic formulations with depigmenting, anti-aging, and regenerative effects. Through microencapsulation, their stability and bioavailability in cosmetic products are enhanced, enabling more effective performance [26,27]. Nowadays, not only natural but also synthetic cinnamic acid derivatives are used as active ingredients of cosmetic products. They still serve as fragrance ingredients but are also used for more specific purposes [28]. Some cinnamic acid derivatives are used as UV filters in cosmetic products [29] and have found use as functional materials—photo-cleavable surfactants [30]. Recent studies have demonstrated the therapeutic properties of cinnamic acid and its derivatives in treating various dermatoses due to their antioxidant, anti-microbial, and anti-inflammatory properties. A reduction in oxidative stress may improve the function of the skin’s hydrolipid barrier and regulate melanin production in conditions such as atopic dermatitis and hyperpigmentation [27,31,32]. Anti-inflammatory effects may benefit in the treatment of acne vulgaris by modulating the inflammatory response associated with the proliferation of *C. acnes* [33]. In cases of contact dermatitis, cinnamic acid derivatives may alleviate inflammation triggered by irritating substances or allergens. However, it is important to note that some cinnamic acid derivatives may act as allergens themselves, potentially inducing contact dermatitis in sensitive individuals [34].

The aim of this article is to present the therapeutic properties of cinnamic acid and its derivatives based on their mechanisms of action in supporting the treatment of the most common dermatoses.

## 2. The Role of Cinnamic Acid in Reducing Oxidative Stress, a Key Factor Inducing Molecular Pathways Involved in the Pathogenesis of Dermatoses

Oxidative stress is a state where the balance between the production of reactive oxygen species (ROS), such as hydrogen peroxide, organic peroxides, and hydroxyl radicals, and the cells’ ability to neutralise these substances is disturbed in favour of ROS production. This is one of the key mechanisms of skin aging, which leads to the degradation of collagen and elastin, causing a loss of skin elasticity and density. ROS attack proteins, lipids, and cellular DNA, accelerating the aging process [35]. In acne vulgaris, there is an observed increase in the production of ROS in response to excessive sebum production and the action of *C. acnes* [4]. This leads to the peroxidation of lipids, especially squalene, with consequent damage of keratinocytes and increased inflammation [5,36]. Oxidative stress can activate signalling pathways leading to the production of pro-inflammatory cytokines and the intensification of inflammation. In inflammatory states, immune cells produce ROS as part of their defence against infections. However, excessive ROS production can lead to tissue damage and increased inflammation [37,38]. In the case of atopic dermatitis, the increased oxidative stress appears as a consequence of the under-activity of antioxidant enzymes, such as superoxide dismutase, catalase, or glutathione peroxidase. Free radicals generated by an excessive immune response further damage the hydrolipid barrier and increase inflammation [39]. Oxidative stress also plays an important role in melanogenesis process. Excessive reactive oxygen species generated by exposure to UV radiation, environmental pollutants, or skin inflammation can directly stimulate melanocytes to overproduce melanin [40].

Cinnamic acid derivatives exhibit high antioxidant activity due to presence of a vinyl fragment and a phenyl ring. Moreover, the presence of hydroxyl or catechol groups in the phenyl ring enhances their antioxidant properties. Dihydroxy derivatives show stronger antioxidant activity compared to monohydroxy derivatives, due to their two hydroxyl groups in the phenyl ring, which significantly improves their ability to neutralise free radicals. The antioxidant properties of cinnamic acid derivatives are mainly associated with the inhibition of lipid oxidation and scavenging of free radicals [31,32].

**Figure 2:**
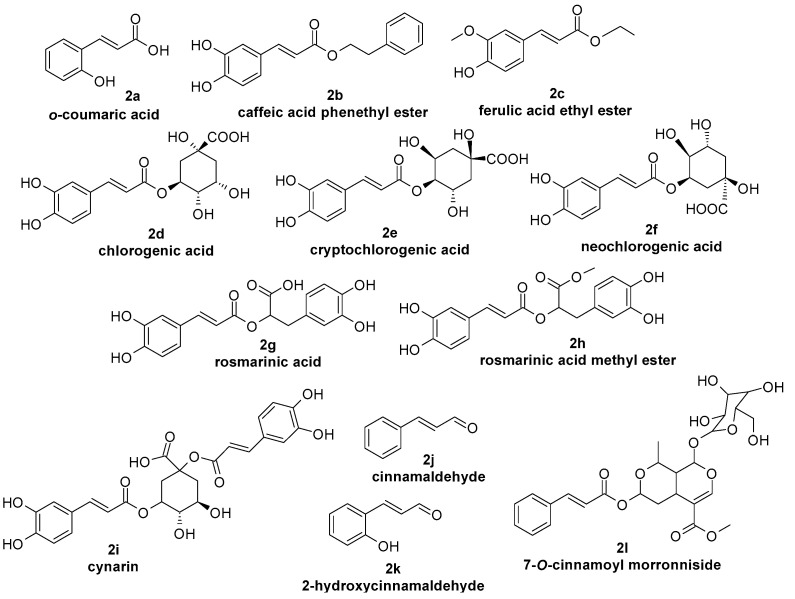
Selected cinnamic acid derivatives with antioxidant and anti-inflammatory properties.

*p*-coumaric acid (Figure 1, **1b**) has a hydroxyl group in the para position relative to the carboxyl group, which acts as an electron donor; thus, it possesses strong antioxidant properties and the ability to neutralise free radicals, which play an important role in the therapy of atopic dermatitis [39]. Due to a more exposed hydroxyl group, *p*-coumaric acid has higher biological activity than its isomer, *o*-coumaric acid (Figure 2, **2a**). The hydroxyl group in the ortho position more readily forms intramolecular hydrogen bonds, which stabilise the molecule but limit its reactivity and bioavailability [41]. Due to its antioxidant action, *p*-coumaric acid can inhibit the signalling pathways linked to gene expression of tyrosinase responsible for the process of melanogenesis and the formation of hyperpigmentation, including PIH [42]. Despite its valuable properties, it is not a common ingredient in cosmetic formulations; it is most often found in the form of extracts from *Panax ginseng*, which are used in anti-aging formulations [18].

Caffeic acid (Figure 1, **1c**) lowers ROS levels and provides significant protection against oxidative stress by scavenging free radicals, which plays a key role in the pathogenesis of the mentioned dermatoses. Due to its two hydroxyl groups in the phenyl ring, caffeic acid is able to form strong hydrogen bonds and donate protons, making it very effective in neutralising free radicals. Those hydroxyl groups also enhance the compound’s antioxidant capacity by stabilising the phenoxyl radical formed after scavenging ROS [43].

Ferulic acid (Figure 1, **1d**) inhibits the oxidation of low-density lipoproteins (LDLs), what helps prevent oxidative damage to cells and tissues. The combination of a hydroxyl group in the para position and a methoxy group in the meta position enhances ferulic acid’s ability to scavenge free radicals making it useful in supporting the therapy of the mentioned dermatoses. The methoxy group increases the lipophilicity of the compound, improving its skin permeation and making it more effective in antioxidant defence, particularly in cosmetic applications [44].

Sinapic acid (Figure 1, **1e**) scavenges free radicals such as DPPH and ABTS, reduces ROS levels, and lowers oxidative stress, protecting cells from oxidative damage. This is due to the presence of two methoxyl groups in the meta positions, which increases the molecule’s ability to donate electrons and stabilises the aromatic ring. A hydroxyl group in the para position allows the molecule to capture free radicals and reduce oxidative stress [45,46].

Caffeic acid phenethyl ester (Figure 2, **2b**) inhibits the formation of superoxide anions and quenches DPPH radicals, providing robust protection against oxidative damage. Esterification of caffeic acid increases the lipophilicity of the molecule and improves bioavailability. The dihydroxyl grouping on the phenyl ring contributes to high radical scavenging activity similar to that of caffeic acid, but with better permeability through the stratum corneum due to the ester bond [47].

Ferulic acid ethyl ester (Figure 2, **2c**) has lower cell-free antiradical capacity than ferulic acid; however, it is more effective in neutralising ROS produced by activated leukocytes compared to ferulic acid. The neutralisation of ROS in areas of skin inflammation associated with the discussed dermatoses is critical for preventing further tissue damage and mitigating the progression of inflammatory processes [48].

Chlorogenic acid (Figure 2, **2d**) scavenges free radicals, particularly DPPH and superoxide anions, protecting cells from oxidative stress and reducing ROS levels. The ester bond between caffeic acid and quinic acid enhances chlorogenic acid’s solubility and bioavailability, increasing its ability to scavenge free radicals. The hydroxyl groups in the caffeic acid moiety contribute to its antioxidant properties by enabling hydrogen donation and the stabilisation of free radicals [49]. Its antioxidant activity, particularly against lipid oxidation, can be utilised to treat acne vulgaris [50].

Cryptochlorogenic acid (Figure 2, **2e**) reduces the production of ROS and increases the activity of antioxidant enzymes such as SOD [51], which can promote regeneration and reduce skin damage, which is important not only in the discussed dermatoses but also in daily skin care [52].

Neochlorogenic acid (Figure 2, **2f**) exhibits strong antioxidant properties, scavenging free radicals and reducing oxidative stress caused by UVB exposure. This helps protect skin cells, such as fibroblasts and keratinocytes, from UV-induced damage, which is especially important in supporting PIH treatment. It also increases the expression of filaggrin, involucrin, loricrin, and caspase-14, which are responsible for the structural integrity of the skin and its hydration, which is crucial in the treatment of atopic dermatitis [53].

Rosmarinic acid (Figure 2, **2g**) reduces lipid peroxidation, decreases NO levels, and enhances antioxidant enzyme activity, including GSH-Px, GSH-Rd, and catalase. The ester bond between caffeic acid and 3,4-dihydroxyphenyl-lactic acid increases the stability of the molecule, while the numerous hydroxyl groups contribute to its strong antioxidant properties. These groups are crucial for hydrogen donation, making rosmarinic acid effective in neutralising ROS and inhibiting lipid peroxidation [54].

Rosmarinic acid methyl ester (Figure 2, **2h**) induces HO-1 expression via Nrf2 activation, which contributes to the reduction in ROS levels [55].

Cynarin (Figure 2, **2i**) reduces ROS levels, scavenges DPPH and ABTS radicals, chelates Fe^2+^ ions, and inhibits lipid peroxidation, protecting cells from oxidative stress. The ester bond in cynarin increases its lipophilicity, allowing the molecule to interact more effectively with lipid radicals. Phenolic hydroxyl groups effectively neutralise free radicals [56]. Cynarin increases the activity of the Nrf-2 pathway, leading to an increase in HO-1 expression and a reduction in cellular ROS levels [57]. Thanks to its strong antioxidant properties, cynarin has the potential to reduce skin damage from acne, atopic dermatitis, or contact allergies. In addition to indirectly affecting melanogenesis (by reducing oxidative stress), it also shows the ability to inhibit tyrosinase, so it can neutralise post-inflammatory hyperpigmentation [58].

Cinnamaldehyde (Figure 2, **2j**) scavenges free radicals such as DPPH and superoxide radicals, reducing the release of ROS and offering protection against oxidative stress. These properties are due to the presence of an aldehyde group, with electrophilicity, allowing the molecule to effectively interact with ROS and scavenge free radicals which are often involved in inflammatory responses [59,60].

## 3. The Role of Cinnamic Acid in Reducing the Inflammatory Response Present in the Course of Dermatoses

Inflammatory reactions play a crucial role in the pathogenesis of both atopic dermatitis (AD) and acne vulgaris, but through different mechanisms. Research on atopic dermatitis has shown the significant role of cytokines, including TNF-α, IL-4, IL-13, and IL-31, in the pathogenesis and symptoms of this condition. TNF-α, produced by various cells such as monocytes, macrophages, and keratinocytes, stimulates the production of reactive oxygen species and pro-inflammatory cytokines, contributing to the exacerbation of skin inflammation. IL-13 and IL-4 are responsible for inducing intercellular oedema and promoting allergic inflammatory reactions, leading to dysfunction of the skin barrier and increased susceptibility to bacterial infections, including *Staphylococcus aureus*. IL-31 enhances the secretion of pro-inflammatory cytokines and chemokines in keratinocytes of patients with AD. Increased expression of the IL-31RA receptor on sensory neurons may contribute to the sensation of itching, a characteristic symptom of AD [9,61].

The inflammatory response in acne vulgaris is triggered by the colonisation of the skin by *Cutibacterium acnes*, leading to the activation of TLR2 receptors on monocytes and keratinocytes. This results in the release of pro-inflammatory cytokines, such as IL-12 and IL-8. In addition, alterations in lipid composition, particularly lipid peroxides, intensify the inflammatory process by promoting keratinocyte proliferation, which contributes to the formation of acne lesions [33,62].

In contact dermatitis, the type of inflammatory response depends on whether the condition is caused by an irritant or an allergen. In irritant contact dermatitis, the skin is directly damaged by a substance, leading to the release of cytokines such as IL-1 and TNF-α, which cause local redness, swelling, and irritation. In allergic contact dermatitis, the immune system is activated by the allergen, causing a delayed reaction. This involves cytokines, which intensify the inflammation, causing symptoms where the allergen made contact with the skin [10,11].

While hyperpigmentation is not primarily an inflammatory condition, inflammation plays a significant role in post-inflammatory hyperpigmentation. PIH can be observed in many skin conditions including acne, eczema, and contact dermatitis. These skin conditions trigger excessive melanin production as part of the healing process. Inflammatory cytokines stimulate melanocytes to overproduce melanin, resulting in dark spots in areas where the skin was previously inflamed [2].

The anti-inflammatory properties of cinnamic acid derivatives may alleviate inflammatory symptoms and support the treatment of the discussed dermatoses. Cinnamic acid derivatives exhibit notable anti-inflammatory activity, mostly due to their ability to modulate key pro-inflammatory pathways (Figure 3). The presence of functional groups such as hydroxyl, methoxy, or ester bonds in these molecules often enhances their anti-inflammatory effects by interacting with various inflammatory mediators [63].

Ferulic acid’s (Figure 1, **1d**) methoxy group improves its interaction with cell membranes and facilitates better inhibition of inflammatory enzymes like COX-2 and iNOS, which is crucial especially in acne vulgaris treatment [64]. The hydroxyl group increases the molecule’s ability to form hydrogen bonds, allowing ferulic acid to effectively inhibit key inflammatory enzymes and modulate the NF-κB pathway, hence reducing the production of pro-inflammatory mediators such as TNF-α, IL-6, and NO [65]. Thus, it can help reduce the inflammatory response in atopic dermatitis, thereby improving skin barrier function [66].

Sinapic acid’s (Figure 1, **1e**) methoxy groups increase the lipophilicity of the molecule enabling modulation of pro-inflammatory enzymes, while the hydroxyl group enables the formation of hydrogen bonds with enzymes leading to effective inhibition of inflammatory processes. As a result, sinapic acid inhibits the production of NO, prostaglandin E2 (PGE2), TNF-α, and IL-1β. In addition, it inhibits the activity of IκB kinase (IKK), which blocks the NF-κB signalling pathway, a major regulator of inflammation, thus reducing the expression of pro-inflammatory genes [67,68]. The immunosuppressive effect of sinapic acid can, among others, alleviate the symptoms of atopic dermatitis [69].

The two hydroxyl groups of caffeic acid (Figure 1, **1c**) contribute to its ability to reduce the inflammatory response by modulating the expression of key cytokines and enzymes involved in the inflammation process. Caffeic acid inhibits inflammatory mediators such as COX-2, IL-8, and NF-κB. Thus, it can contribute to a reduction in the symptoms of the dermatoses in discussion [70,71].

Chlorogenic acid (Figure 2, **2d**) decreases the expression of TNF-α, IL-1β, IL-6, IL-8, TLR-2/4, and MMP2/9 by reducing the activity of the NF-κB signalling pathway in epidermal cells. Therefore, it may be used as a potential anti-acne agent targeting inflammation [72].

Cryptochlorogenic acid (Figure 2, **2e**) inhibits the nuclear translocation of NF-κB, leading to the suppression of pro-inflammatory cytokines. It also suppresses the production of NO, TNF-α, and IL-6, which are key pro-inflammatory mediators. Thus, cryptochlorogenic acid can support the treatment of acne vulgaris, atopic dermatitis, or contact dermatitis by reducing inflammatory reactions [51].

Caffeic acid phenethyl ester (Figure 2, **2b**) can more easily interact with inflammatory enzymes due to its ester bond. It inhibits LOX and COX, which are involved in the arachidonic acid metabolism pathway and are directly responsible for the inflammatory processes found in atopic dermatitis [73,74].

Ferulic acid ethyl ester (Figure 2, **2c**) acts as an NADPH oxidase inhibitor, thereby reducing ROS production. This effect is beneficial in dermatoses associated with oxidative stress and chronic inflammation [48].

Rosmarinic acid’s (Figure 2, **2g**) hydroxyl groups are responsible for its interaction with inflammatory enzymes like MMP-9 and for modulating the NF-κB pathway [75]. This results in decreasing expression of pro-inflammatory mediators and reducing inflammation, both in acne vulgaris and atopic dermatitis [76,77]. Rosmarinic acid also reduces skin inflammation and itching in allergic contact dermatitis by inhibiting the MRGPRX2/PLCγ1 signalling pathway [78].

Rosmarinic acid methyl ester (Figure 2, **2h**) inhibits NO production by suppressing iNOS expression. It also inhibits both the upstream and downstream pathways of IFN-β production, which is activated in response to *C. acnes* and *Malassezia* infection [55]. This occurs in the course of acne vulgaris. Rosmarinic acid methyl ester can alleviate the symptoms of an excessive inflammatory response [79].

2-Hydroxycinnamaldehyde (Figure 2, **2k**) inhibits NO production and reduces COX-2 and TNF-α activity, contributing to its antioxidant and anti-inflammatory effects. This is due to the presence of a hydroxyl group in position 2 of the phenyl ring, which increases the molecule’s ability to donate hydrogen atoms, increasing its radical scavenging potential. The hydroxyl group also allows stronger interactions with enzymes and receptors involved in inflammation [60]. It has been shown to mitigate inflammatory responses, barrier damage, and apoptosis by inhibiting STAT3 activation, so it may be useful in treating the dermatoses discussed [80].

Cynarin (Figure 2, **2i**) has an ester bond, which allows it to better interact with lipid membranes. In turn, hydroxyl groups, in addition to reducing ROS, are also responsible for modulating enzymes. This has a direct effect on alleviating inflammation by inhibiting the p38 MAPK and NF-κB pathways, as well as inhibiting iNOS, which is important in calming dermal inflammation. [81].

7-O-Cinnamoyl morronniside (Figure 2, **2l**) reduces inflammation by inhibiting TNF-α-induced E-selectin expression, which plays a critical role in the adhesion of leukocytes to endothelial cells, a process crucial to the inflammatory response. This is due to the presence of a cinnamate moiety and an ester bond, which increase lipophilicity, allowing the compound to permeate cell membranes and interact with cytokines [32,82]. Inhibition of TNF-α-induced E-selectin expression is crucial in alleviating the symptoms of inflammatory skin diseases, particularly atopic dermatitis [83].

Cinnamaldehyde (Figure 2, **2j**) at low concentrations (100 µM) decreases the expression of pro-inflammatory cytokines, including IL-1β, IL-6, IL-8, and TNF-α. However, at high concentrations (250 µM), it increases the expression of these pro-inflammatory cytokines and induces increased apoptosis of keratinocytes, potentially exacerbating the inflammatory response [84].

## 4. Cinnamic Acid Derivatives as Antimelanogenic Agents

Potential targets for depigmenting agents include various mechanisms, such as inhibiting melanocyte stimulation, preventing melanosome transfer, or breaking down melanin within keratinocytes, but most commonly used agents primarily inhibit tyrosinase. Tyrosinase is a metalloenzyme from the oxidoreductase group, found in humans, animals, plants, fungi, and bacteria, with a molecular weight of 60 to 80 kDa. Its active site possesses two copper centers, CuA and CuB, essential for its catalytic function. Tyrosinase shows monophenolase activity (converting L-tyrosine to L-DOPA) and diphenolase activity (converting L-DOPA to L-dopaquinone). It has three domains: the *N*-terminal, responsible for localisation signals and proenzyme activation, the catalytic domain containing two copper ions for redox reactions, and the C-terminal responsible for structure stabilisation and protein interactions. Tyrosinase is a rate-limiting enzyme in melanogenesis, initiating the conversion of tyrosine into dopaquinone, a precursor in melanin production. During inflammatory conditions or trauma, the activation of melanocytes is driven by pro-inflammatory cytokines and oxidative stress, which upregulate tyrosinase activity and lead to excessive melanin synthesis [88].

The (*E*)-β-phenyl-α,β-unsaturated carbonyl group plays a key role in the potent inhibition of tyrosinase activity. Cinnamic acids, which possess this moiety, have not attracted attention as potential tyrosinase inhibitors due to their relatively low tyrosinase inhibitory activity and relatively high hydrophilicity. The presence of hydroxyl groups—their number and position in the molecule—affect the tyrosinase inhibitory activity; the carboxyl group, which has a high polarity, hinders the penetration of the substance into the basal layer of the epidermis, where melanocytes are located; so, during the synthesis of cinnamic acid, derivatives begin to replace the carboxyl group with other functional groups of lower polarity. For example, an amide group which is stable and shows good pharmacological properties also exhibits promising tyrosinase inhibitory properties [89].

Understanding the types of enzyme inhibition, which involve mechanisms where inhibitors bind to enzymes and reduce their activity, is crucial for designing new compounds with high efficacy and selectivity [90]. Several types of enzyme inhibition are distinguished. In competitive inhibition, the inhibitor bears structural similarity to the enzyme’s substrate and competes for binding at the active site of the enzyme. The enzyme binds to the inhibitor instead of the substrate, forming a complex that is catalytically inactive. Increasing the substrate concentration can reduce the number of enzyme–inhibitor complexes. In uncompetitive inhibition, the inhibitor binds exclusively to the enzyme–substrate complex, forming an enzyme–substrate–inhibitor complex that is catalytically inactive. The uncompetitive inhibitor does not bind to the free enzyme [91]. In non-competitive inhibition, the inhibitor does not exhibit structural similarity to the substrate and binds to the enzyme at a site other than the active site. The inhibitor can bind to both the free enzyme and the enzyme–substrate complex with equal affinity, forming catalytically inactive complexes. In mixed inhibition, the inhibitor can bind to either the free enzyme or the enzyme–substrate complex but with different affinities. The inhibitor binds at a site other than the active site, affecting both the formation of the enzyme–substrate complex and its conversion to the product [92]. These mechanisms are critical for the development of drugs that target specific enzymes, allowing for the design of inhibitors with desired properties. Various cinnamic acid derivatives were identified as tyrosinase inhibitors; the type of inhibition identified for them is summarised in Table 1.

**Table 1:** Type of tyrosinase inhibition identified for selected cinnamic acid derivatives.

Compound	Type of Inhibition	
	Monophenolase	Diphenolase
Cinnamic acid Figure 1, 1a		Mixed [91] Non-competitive [93]
*p*-coumaric acid Figure 1, 1b	Non-competitive [94,95] Mixed [96]	Competitive [93,96,97] Mixed [96,98]
*o*-coumaric acid Figure 2, 2a		Competitive [99]
Ferulic acid Figure 1, 1d		
4-Methoxycinnamic acid Figure 4, 4a	Non-competitive [95]	Non-competitive [93,100] Mixed [98]
*p*-coumaric acid ethyl ester Figure 4, 4b	Non-competitive [101]	Competitive [102]
4-Chlorocinnamic acid Figure 4, 4c		Non-competitive [103]
Cinnamaldehyde Figure 2, 2j		Non-competitive [98,100]

**Figure 4:**
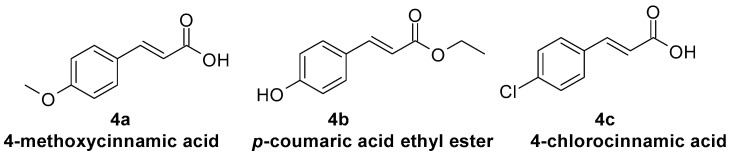
Selected cinnamic acid derivatives with tyrosinase inhibitory properties.

Cinnamic acid was also tested in the melan-a mouse melanocyte cell line and showed an inhibitory effect on melanin production and tyrosinase activity (IC_50_ = 693.2 µM) [104]. Caffeic acid *N*-nonyl ester was proved to inhibit diphenolase activity of mushroom tyrosinase with an IC_50_ of 37.5 µM; on the contrary, caffeic acid *N*-heptyl ester showed no tyrosinase inhibitory activity [105]. It was also shown to inhibit melanin synthesis in B16F10 cells (at concentrations 5–50 μM), and it was proposed as an active ingredient from *Rhodiola sachalinensis* as well as from *Sasa quelpaertensis* for the inhibition of melanogenesis [106,107]. It also showed a potent antimelanogenic effect in human epidermal melanocytes (HEM) exposed to UVB in which it was able to significantly reduce melanin content [96]. *Pulsatilla cernua* root-derived materials were proved to inhibit mushroom tyrosinase; the bioactive components of the used extracts were identified, including caffeic acid and ferulic acid [9]. It was proposed that *p*-coumaric acid inhibits tyrosinase due to the similarity of its chemical structure to L-tyrosine. Studies have shown that it may be a stronger inhibitor of human and murine tyrosinase than mushroom tyrosinase [96]. However, recent findings showed that cinnamic acid, *o*-hydroxycinnamic acid, and 2-, 3-, and 4-methoxycinnamic acid are tyrosinase inhibitors, but *p*-coumaric acid as well as 3-hydroxycinnamic acid act as substrates of tyrosinase, which catalyses their hydroxylation resulting in the formation of corresponding *o*-quinones [99,108]. Caffeic acid and caffeic acid *N*-nonyl ester also act as suicide substrates of the enzyme due to their *o*-diphenolic structure [108,109]. These findings were confirmed in kinetic, computational, and spectroscopic studies.

**Figure 5:**
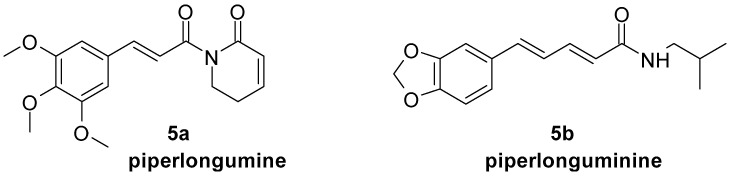
Chemical structures of piperlongumine and piperlonguminine.

Piperlongumine (Figure 5, **5a**), an alkaloid extracted from *Piper* plants, especially the roots of *Piper longum*, is an amide derivative of cinnamic acid [110]. It showed melanogenesis inhibition properties in B16F10 cells in vitro (at 3 and 6 μM) as well as in zebra fish embryos in vivo (at 2 μM). It also inhibited the expression of melanogenesis-related genes in B16F10 cells such as *Mitf*, microphthalmia-associated transcription factor; *Tyr*, tyrosinase; *Trp-1*, tyrosinase-related protein 1; and *Trp-2*, tyrosinase-related protein 2 [111]. Another alkaloid from *Piper longum*, piperlonguminine (Figure 5, **5b**), possesses an extended linker between the phenyl ring and amide group. It showed an inhibitory effect on melanin production in the B16F10 cell line by downregulation tyrosinase expression at concentrations as low as 6 μM [112]. 

Several synthetic amide derivatives of cinnamic acid, with various substituents in the phenyl ring as well as in the amide group also showed promising anti-melanogenesis activity. The examples of active compounds in that group are presented in Table 2.

**Figure 6:**
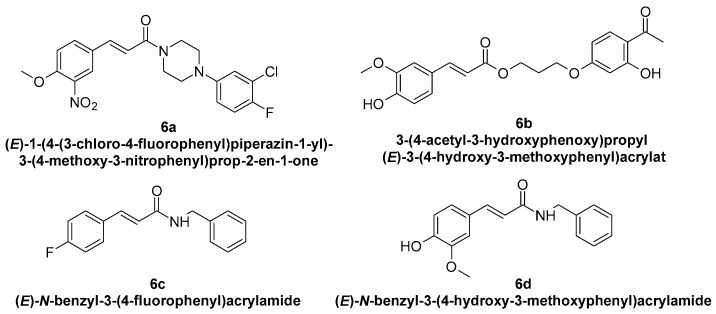
Chemical structures of synthetic cinnamic acid derivatives with proved tyrosinase inhibitory properties [117,118,120].

Compounds with hydroxyl groups in the phenyl ring (Table 2) were also tested for antioxidant activity in DPPH assays and proved promising properties. Antioxidant activity may contribute to melanogenesis inhibition [89,113,114,115,116]. Some of the presented compounds (Table 2) were also tested in more advanced models. (*E*)-3-(4-chlorophenyl)-*N*-(5-hydroxypentyl)acrylamide was shown to penetrate through reconstructed human epidermis and inhibit melanin biosynthesis in MelanoDerm (pigmented reconstructed human epidermis) tissue [116]. Moreover, several synthetic derivatives were also proved to inhibit melanogenesis, e.g., (*E*)-1-(4-(3-chloro-4-fluorophenyl)piperazin-1-yl)-3-(4-methoxy-3-nitrophenyl)prop-2-en-1-one (Figure 6, **6a**) is a mushroom tyrosinase inhibitor, and it inhibits tyrosinase activity in A375 human melanoma cells (at 25 and 50 µM) as well as melanogenesis in zebrafish in in vivo assays (at 25 and 50 µM) [117]. Synthetic ester derivatives of cinnamic acid were also found to inhibit melanogenesis. One of the most promising compound, 3-(4-acetyl-3-hydroxyphenoxy)propyl (*E*)-3-(4-hydroxy-3-methoxyphenyl)acrylate (Figure 6, **6b**), inhibited mushroom tyrosinase activity by 82.4 ± 1.36% when tested at 1 mM and also inhibited cellular tyrosinase activity in B16F10 cells in a concentration range of 25–200 μM [118]. *p*-hydroxycinnamic acid isopropyl ester was shown not only to inhibit tyrosinase but also to reduce the melanin deposition in human skin fragments at a 0.1% concentration. In this case, esterification let to an important equilibrium between tyrosinase affinity and lipophilicity to allow adequate permeability in the skin [119]. (*E*)-*N*-Benzyl-3-(4-hydroxy-3-methoxyphenyl)acrylamide (Figure 6, **6c**) and (*E*)-*N*-benzyl-3-(4-fluorophenyl)acrylamide (Figure 6, **6d**) showed antimelanogenic activity in normal human epidermal melanocytes (NHEMs) as well as in a 3D model comprising NHME and primary normal human epidermal keratinocytes (NHEK) [120].

## 5. Cinnamic Acid Derivatives Exhibiting Antimicrobial Activity Against Microorganisms Associated with Specific Dermatoses

In the case of the dermatoses described earlier such as acne vulgaris and atopic dermatitis, dysregulation of the skin microbiota is observed. This makes it easier for opportunistic microorganisms to cause infections [36]. A crucial approach in their treatment is targeting both antibacterial and antifungal activities.

In Annex V of the Cosmetic Regulation (EC) No 1223/2009 [121], which provides a list of permitted preservatives, neither cinnamic acid nor any of its derivatives are specified. However, cinnamic acid is present in the cosmetic market as an anti-microbial agent, preservative, and rpeservative booster (manufacturer: Cosphatec, Hamburg, Germany, raw material: Cosphaderm^®^ CA natural). The manufacturer claims that cinnamic acid can replace “controversial” preservatives such as parabens [122].

In acne vulgaris and atopic dermatitis, the skin is often colonised by bacteria such as *Cutibacterium acnes*, *Staphylococcus aureus*, *Staphylococcus epidermis*, *Candidia* spp., and *Malassezia* spp., which can enhance clinical symptoms. Failure to adhere to microbiological standards in cosmetics can further worsen skin conditions by promoting the growth of pathogens. Due to their antimicrobial properties, cinnamic acid derivatives constitute potential candidates for the treatment of skin disorders caused by microbial infections. They are also promising candidates for incorporation into cosmetic formulations, as they can simultaneously alleviate the symptoms of acne vulgaris or atopic dermatitis while ensuring compliance with microbiological safety standards [85,123].

The primary mechanism of the antimicrobial activity of phenolic compounds is directly associated with the disruption of microbial membranes, enzyme interaction, or metal ion deprivation. The main cause of the antibacterial activity of cinnamic acid derivatives is the presence of the α,β-unsaturated carbonyl group in their structure, which is highly reactive and causes covalent modification of key bacterial proteins, such as NADH oxidoreductases, ABC transporters, and ATP-binding proteins, leading to cell destabilisation and death [124]. Cinnamic acid (Figure 1, **1a**) exhibits weak antibacterial activity against most Gram-negative and Gram-positive bacteria, as well as antifungal activity. It showed MIC values of 1000 µg/mL against *Staphylococcus epidermis* and 18–20 nM against *Staphylococcus aureus.*

**Figure 7:**
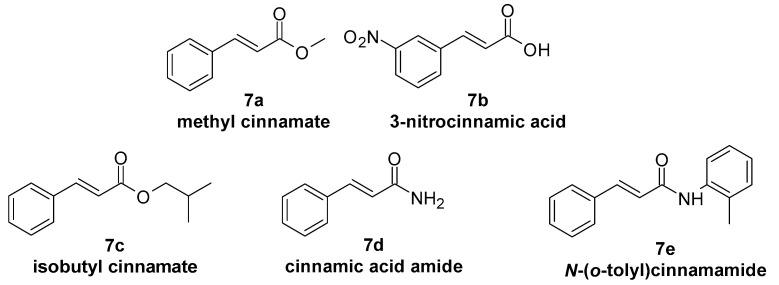
Selected cinnamic acid derivatives with antimicrobial activity against microorganisms associated with dermatoses.

Antimicrobial activity was also proved among others for the following:

- Ferulic acid (Figure 1, **1d**) against *Staphylococcus aureus* [125] and *Candida albicans* [126];
- Sinapic acid (Figure 1, **1e**) against *Staphylococcus aureus* [127];
- Caffeic acid (Figure 1, **1c**) against *Staphylococcus epidermis* [128] and *Staphylococcus* [129];
- Chlorogenic acid (Figure 2, **2d**) against *Staphylococcus aureus* [130], *Malassezia*, and *Candida albicans* [131];
- Rosmarinic acid (Figure 2, **2g**) against *Staphylococcus aureus* [132], *Staphylococcus epidermis* [133], and *Candida albicans* [134];
- 4-methoxycinnamic acid (Figure 4, **4a**) against *Staphylococcus aureus* and *Candida albicans* [135];
- Methyl cinnamate (Figure 7, **7a**) against *Candida albicans* [136], *Staphylococcus epidermis*, and *Staphylococcus aureus* [135];
- 3-nitrocinnamic acid (Figure 7, **7b**) against *Candida albicans* and *Staphylococcus aureus* [135];
- Isobutyl cinnamate (Figure 7, **7c**) against *Candida albicans* and *Staphylococcus aureus* [135];
- Cinnamamide (Figure 7, **7d**) against *Candida albicans* and *Staphylococcus aureus* [135];
- *N*-(*o*-tolyl)cinnamamide (Figure 7, **7e**) against *Candida albicans* and *Staphylococcus aureus* [135];
- Cinnamaldehyde (Figure 2, **2j**) against *Staphylococcus epidermidis* [137] and *Cutibacterium acnes* regarding oleum cinnamomi, the main component of which is cinnamaldehyde) [124].

## 6. Contact Dermatitis—Therapeutic Area and Dermatological Concern Caused by Selected Cinnamic Acid Derivatives

**Figure 8:**
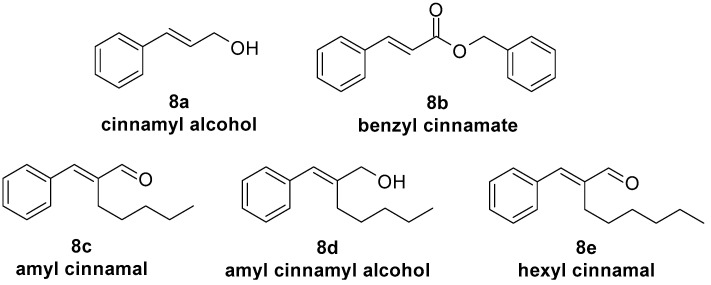
Chemical structures of cinnamic acid derivatives used as fragrance materials.

Contact dermatitis is a localised skin reaction characterised by redness and itching after contact with a foreign substance. Contact dermatitis can be caused by irritation or an allergic reaction [138]. Irritation results from direct, non-immunologic damage to the skin by a substance, manifesting quickly after application and resolving after its removal. The main pathophysiological changes include disruption of the skin barrier, alterations in epidermal cells, and the release of proinflammatory cytokines [139]. Allergic contact dermatitis is a delayed hypersensitivity reaction, with symptoms potentially appearing 24–48 h after exposure to the allergen and persisting even after the product is removed, affecting both the site of application and distant areas. Contact allergy is a specific hypersensitivity of the body to low-molecular-weight substances or proteins, triggered by direct contact of these substances with the skin. [11]. Selected cinnamic acid derivatives with anti-inflammatory activity could possibly alleviate inflammation triggered by irritating substances or allergens. However, some compounds from that group may cause contact dermatitis. Among the ingredients found in cosmetics, contact hypersensitivity is most commonly triggered by preservatives, fragrances, and colorants [10]. *Cinnamomum cassia* leaf oil, *Cinnamomum zeylanicum* bark oil, and cinnamic acid derivatives, such as cinnamaldehyde (Figure 2, **2j**), cinnamyl alcohol (Figure 8, **8a**), benzyl cinnamate (Figure 8, **8b**), amyl cinnamal (Figure 8, **8c**), amyl cinnamyl alcohol (Figure 8, **8d**), and hexyl cinnamal (Figure 8, **8e**), are used to provide fragrance in cosmetic products. Since they are classified as potential allergens, they are listed in Annex III of Regulation (EC) No 1223/2009 [121] on cosmetic products. They may cause contact dermatitis.

## 7. Conclusions

Cinnamic acid and its derivatives, both natural and synthetic, may be used in cosmetic products supporting the treatment of selected dermatoses such as acne vulgaris, atopic dermatitis, contact dermatitis, and hyperpigmentation. The mechanisms of action of cinnamic acid derivatives involve antioxidant, anti-inflammatory, and antimicrobial properties, as well as antimelanogenic activity. Neutralisation of reactive oxygen species results in a reduction in oxidative stress, which may improve the function of the skin’s hydrolipid barrier and regulate melanin production in atopic dermatitis and hyperpigmentation. Additionally, anti-inflammatory effects may benefit the treatment of acne vulgaris by modulating the inflammatory response associated with the proliferation of *Cutibacterium acnes*. In cases of contact dermatitis, cinnamic acid derivatives may alleviate inflammation triggered by irritating substances or allergens. This complex molecular mechanism is the cause of the multifunctional potential of selected derivatives which can be used in as active ingredients in cosmetic formulations. Especially promising multifunctional compounds are cinnamic acid, *p*-hydroxycinnamic acid, ferulic acid, sinapic acid, and caffeic acid which exert more than one molecular mechanism of action. This multifunctional action is particularly important in the treatment of acne vulgaris and atopic dermatitis. However, despite the numerous benefits of their application, it is also important to consider the potential risk of allergic reactions or irritation caused by cinnamic acid derivatives. Therefore, when designing new cosmetic formulations, it is essential to minimise any possible adverse effects.

## Figures and Tables

**Table 2:** Melanogenesis inhibitors in the group of synthetic cinnamic acid amide derivatives.

Compound	Structure	Mushroom Tyrosinase Inhibition [% ± SD]/Tested Concentration	Concentration Showing Melanin Synthesis Inhibition in A-MSH-Stimulated B16F10 Cell	Reference
(*E*)-*N*-(3-(3,4-Dihydroxyphenyl)acryloyl)benzamide	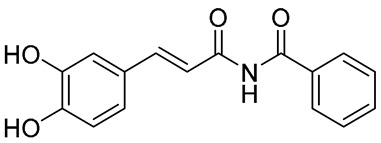	25.99 ± 2.77/25 μM	25 μM	[113]
(*E*)-3-(2,4-Dihydroxyphenyl)-1-(pyrrolidin-1-yl)prop-2-en-1-one	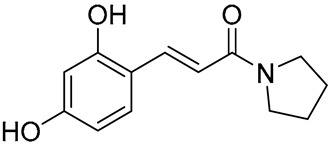	93.72 ± 0.25/25 μM	5 μM	[89]
(*E*)-3-(2,4-Dihydroxyphenyl)-*N*,*N*-diethylacrylamide	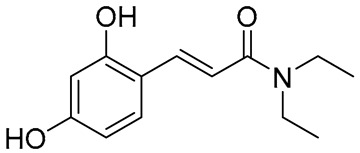	96.20 ± 1.44/25 μM	5 μM	[114]
(*E*)-*N*-Cyclohexyl-3-(2,4-dihydroxyphenyl)acrylamide	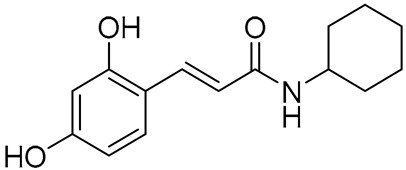	93.78 ± 1.42/25 μM	5 μM	[115]
(*E*)-3-(4-Chlorophenyl)-*N*-(5-hydroxypentyl)acrylamide	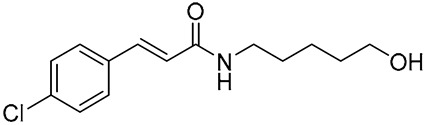	88.98 ± 0.98/500 μM	6.25 μM	[116]
